# Health benefits of pedestrian and cyclist commuting: evidence from the Scottish Longitudinal Study

**DOI:** 10.1136/bmjph-2024-001295

**Published:** 2024-05-15

**Authors:** Catherine Friel, David Walsh, Bruce Whyte, Chris Dibben, Zhiqiang Feng, Graham Baker, Paul Kelly, Evangelia Demou, Ruth Dundas

**Affiliations:** 1 MRC/CSO Social and Public Health Sciences Unit, University of Glasgow, Glasgow, UK; 2 School of Health & Wellbeing, University of Glasgow, Glasgow, UK; 3 Glasgow Centre for Population Health, Glasgow, UK; 4 Institute of Geography, University of Edinburgh, Edinburgh, Lothian, UK; 5 University of Edinburgh, Edinburgh, UK; 6 University of Edinburgh Institute for Sport Physical Education and Health Sciences, Edinburgh, UK

**Keywords:** Public Health, Accidents, Traffic, Epidemiology

## Abstract

**Background:**

Despite active travel investment increasing, evidence of benefit is often limited to selected health outcomes and a short follow-up period, and cyclists and pedestrians are often analysed together. We aimed to examine prospective associations with multiple health outcomes over 18 years for pedestrians and cyclists separately.

**Methods:**

The Scottish Longitudinal Study is based on census data, from which we selected 82 297 individuals aged 16–74 years. Individuals were followed-up between 2001 and 2018 through linkage to hospitalisation, death and prescription records. Cox proportional hazard models were used to compare cyclist and pedestrian commuters with non-active commuters for a range of health outcomes, controlling for pre-existing health conditions, and demographic and socioeconomic characteristics.

**Results:**

Compared with non-active commuting, cyclist commuting was associated with lower all-cause mortality risk (HR 0.53, 95% CI 0.38 to 0.73), lower risk of any hospitalisation (HR 0.90, 95% CI 0.84 to 0.97), lower risk of cardiovascular disease (CVD) hospitalisation (HR 0.76, 95% CI 0.64 to 0.91) and of having a CVD prescription (HR 0.70, 95% CI 0.63 to 0.78), lower risk of cancer mortality (HR 0.49, 95% CI 0.30 to 0.82) and cancer hospitalisation (HR 0.76, 95% CI 0.59 to 0.98), and lower risk of having a prescription for mental health problems (HR 0.80, 95% CI 0.73 to 0.89). Pedestrian commuting was associated with lower risk of any hospitalisation (HR 0.91, 95% CI 0.88 to 0.93), lower risk of CVD hospitalisation (HR 0.90, 95% CI 0.84 to 0.96) and of having a CVD prescription (HR 0.90, 95% CI 0.87 to 0.93), and lower risk of a mental health prescription (HR 0.93, 95% CI 0.90 to 0.97).

**Conclusion:**

Active commuters were less likely to suffer from a range of negative physical and mental health outcomes than non-active commuters. These findings strengthen the evidence for the health benefits of active commuting.

**WHAT IS ALREADY KNOWN ON THIS TOPIC**

- Cyclist commuting, and to a lesser degree pedestrian commuting, have been associated with a lower risk of morbidity and mortality.
- Evidence of the association between walking and cycling to work and many health outcomes, including mental health, is limited.

**WHAT THIS STUDY ADDS**

- This study, based on a large representative sample followed up for 18 years, provides robust evidence of lower risk of all-cause mortality, any hospital admission, cardiovascular disease hospitalisation and medication, cancer incidence and mortality and medication for poor mental health among cycling commuters compared with non-active commuters.
- Walking to work compared with non-active commuting was associated with reduced risk of any hospitalisation, cardiovascular disease hospitalisation and medication, and medication for poor mental health.

**HOW THIS STUDY MIGHT AFFECT RESEARCH, PRACTICE OR POLICY**

- With an increased focus on active travel within health, environmental and planning policy in Scotland alongside substantial increases in funding, this study provides timely evidence of the health benefits of active commuting for both local, national and international policymakers.

## Introduction

 Regular moderate-to-vigorous physical activity, such as walking and cycling, has multiple physical and mental health benefits.^1 2^ A 2014 systematic review and meta-analysis showed evidence of significant reductions in all-cause mortality associated with both walking (11%) and cycling (10%), while adjusting for other levels of physical activity.^3^

Active travel has been referred to as the most practical and sustainable way to increase daily physical activity,^4^ and there is growing evidence of the health benefits associated with active commuting, principally walking and cycling for work and study.^5^ A 2008 meta-analytic review confirmed the protective effects of active commuting on cardiovascular incidence and mortality.^6^ A large-scale, UK study demonstrated an association between cycle commuting and lower risks of all-cause and cause-specific mortality, while controlling for a broad range of behavioural and biological risk factors.^7^ Despite many strengths, this study had a relatively narrow age range of participants (40–69 years), a short period of follow-up (5 years) and acknowledged a potential for a healthy volunteer selection bias.

Despite the known benefits, existing evidence mostly relates to a limited set of health outcomes such as morbidity and mortality related to cardiovascular disease (CVD), cancer, and all-cause mortality. Better psychological well-being scores have been associated with active commuting^8 9^ and enhanced mental health has been measured in some settings, for example, among people who actively commute through natural environments compared with car commuters.^10^ However, the links between active commuting and mental health and well-being are still unclear^11 12^ and relatively few studies have reported on the independent associations of walking and cycling to work with mental health.^9^ It is important to look at the impacts of walking and cycling separately given their differing prevalence, health impacts, infrastructure requirements and implications for environmental policy.^13^ Linked to this, cycling in cities is increasing,^14^ encouraged by new bike use options, including e-bikes and bike-share schemes,^15^ which are contributing to increased physical activity and a modal shift to more sustainable travel.

In Scotland, levels of walking to work have reduced significantly in the last 50 years^16^ and levels of active commuting remain relatively low.^17^ While active travel investment is now increasing^18^ there is currently only limited indirect evidence in a Scottish context to assess potential long-term health benefits.^19^

The aims of this new study are to address some of the limitations of previous studies, through a longer follow-up period, broader age groups and an expanded set of outcomes: all causes, CVD, cancer, poor mental health, and traffic collision casualties. By using a large representative national sample, this will generate new evidence of relevance to policymakers, nationally and internationally.

The principal research question this study addressed is: how does the risk of various physical and mental health outcomes differ between pedestrian commuters and cyclist commuters versus non-active commuters, over an 18 year period?

## Methods

### Study design

We accessed data from the Scottish Longitudinal Study (SLS), a nationally representative sample based on 5.3% of the Scottish population derived from the Census in 1991, 2001 and 2011.^20^ The SLS sample was linked to national hospital admission and death registration data and prescription information system data using personal identifiers. We chose to focus on 2001 as the base year due to the unavailability of key covariate measures in 1991 and 2011, for example, urban/rural classification and a comparable overcrowding measure in 2011.

### Participants

The population of interest was all participants aged 16–74 years in 2001 who travelled to a place of work or study in the UK, and so excluded the unemployed, offshore workers and those working outside the UK, leaving 114 523 people. Four hundred and sixty-seven records for active commuters travelling distances over 40.5 km were excluded, based on previous research which revealed a minority of pedestrian and cycling commuters who appeared to commute implausibly long distances.^19^ We excluded a further 31 759 individuals with missing covariate data. For most variables, <5% of cases had missing values and the distribution of missingness was similar across modes of travel; exceptions to this were the overcrowding and distance to work variables which had 6.3% and 17.8% missing data, respectively. Thus, the final SLS sample used in the study comprised 82 297 participants.

### Patient and public involvement

Our study used anonymised data held within the Scottish Longitudinal Study. For this reason it was not appropriate to involve patients or the public in the design, or conduct, or reporting or dissemination plans of our research.

### Exposure

The exposure variable was derived from responses to the national Census question: ‘How do you usually travel to your main place of work or study (including school)?’. Respondents were asked to select which mode of travel they used for the longest part, by distance, of their usual journey. Active travel was defined as either on foot (pedestrian) or by bicycle (cyclist). All other modes of commuting were defined as non-active. Thus, the exposure variable was mode of travel to work coded as: non-active, pedestrian or cyclist.

### Covariates

Covariates were: age, sex, pre-existing health condition (defined, based on similar previous studies,^7 21^ as hospitalisation within the 5 years before baseline (2001) for diabetes, cardiovascular disease, depression or cancer), socioeconomic factors (housing tenure, National Statistics Socioeconomic Classification (NS-SEC), highest educational qualification, household overcrowding) and a range of other potential confounders (shift worker status, distance to work from home, urban or rural place of residence, presence or absence of dependent children, carer status). A full list of the covariates and categories, tabulated by mode of commuting, is shown in table 1.

**Table 1:** Covariates and categories tabulated by mode of commuting

	Mode of commuting	Cyclist	Pedestrian	Non-active
Total sample size		1363	11 561	69 373
**Covariates**	**Category**	**n (%n)**	**n (%)**	**n (%)**
Pre-existing health condition*	No	1341 (98.4)	11 284 (97.6)	67 562 (97.4)
	Yes	22 (1.6)	277 (2.4)	1811 (2.6)
Sex	Female	319 (23.4)	7115 (61.5)	35 342 (50.9)
	Male	1044 (76.6)	4446 (38.5)	34 031 (49.1)
Age group (years)	16–29	378 (27.7)	4100 (35.5)	17 510 (25.2)
	30–44	639 (46.9)	3844 (33.2)	28 925 (41.7)
	45–74	346 (25.4)	3617 (31.3)	22 938 (33.1)
Shift worker	No	973 (71.4)	6996 (60.5)	51 488 (74.2)
	Yes	390 (28.6)	4565 (39.5)	17 885 (25.8)
Distance to work	<5 km	1106 (81.1)	11 299 (97.7)	28 843 (41.6)
	5–9.9 km	187 (13.7)	111 (1.0)	15 834 (22.8)
	10–14.9 km	35 (2.6)	35 (0.3)	8720 (12.6)
	15 km or further	35 (2.6)	116 (1.0)	15 976 (23.0)
Highest qualification	No qualification	236 (17.3)	2885 (25.0)	11 714 (16.9)
	Level 1 – ‘O’ Grade/Standard grade/GCSE/CSE etc/GSVQ/SVQ level 1 or 2/SCOTVEC module	336 (24.7)	3292 (28.5)	18 358 (26.5)
	Level 2 – Higher grade /CSYS/‘A’ level, etc/GSVQ /SVQ Level 3/ONC/OND	238 (17.5)	2428 (21.0)	13 007 (18.7)
	Level 3 – HNC/HND/SVQ level 4 or 5	104 (7.6)	722 (6.2)	6959 (10.0)
	Level 4 – First degree/higher degree/professional qualifications	449 (32.9)	2234 (19.3)	19 335 (27.9)
Dependent children	No	797 (58.5)	7354 (63.6)	40 486 (58.4)
	Yes	566 (41.5)	4207 (36.4)	28 887 (41.6)
Carer status	No	1259 (92.4)	10 389 (89.9)	61 323 (88.4)
	Yes	104 (7.6)	1172 (10.1)	8050 (11.6)
Housing tenure	Home owner	995 (73.0)	7340 (63.5)	57 210 (82.5)
	Non-home owner	368 (27.0)	4221 (36.5)	12 163 (17.5)
National Statistics-Socioeconomic Classification (NS-SEC)	1. Higher managerial, administrative and professional occupations	201 (14.7)	738 (6.4)	8558 (12.3)
	2. Lower managerial, administrative and professional occupations	293 (21.5)	1770 (15.3)	19 626 (28.3)
	3. Intermediate occupations	116 (8.5)	1295 (11.2)	10 850 (15.6)
	4. Small employers and own account workers	24 (1.8)	515 (4.5)	2684 (3.9)
	5. Lower supervisory and technical occupations	193 (14.2)	1038 (9.0)	6988 (10.1)
	6. Semi-routine occupations	237 (17.4)	2754 (23.8)	9674 (13.9)
	7. Routine occupations	181 (13.3)	1731 (15.0)	7170 (10.3)
	8. Students	118 (8.7)	1720 (14.9)	3823 (5.5)
Urban-rural classification	Primary city – pop 125 000+	608 (44.6)	4894 (42.3)	25 893 (37.3)
	Urban settlements – pop 10 000+	378 (27.7)	3364 (29.1)	21 240 (30.6)
	Small accessible towns – pop 3000+	140 (10.3)	1158 (10.0)	7585 (10.9)
	Small remote towns – pop 3000+	55 (4.0)	600 (5.2)	1470 (2.1)
	Accessible rural	129 (9.5)	937 (8.1)	9905 (14.3)
	Remote rural	53 (3.9)	608 (5.3)	3280 (4.7)
Overcrowded housing	No	1237 (90.8)	9876 (85.4)	63 596 (91.7)
	Yes	126 (9.2)	1685 (14.6)	5777 (8.3)

* Diabetes (ICD-10 codes: E10-E14, ICD-9 codes: 250.0–250.3) or cardiovascular disease (ICD-10: I00-I99, ICD-9: 390–459) or cancer (ICD-10: C00-C97, ICD-9: 140–208) or depression (ICD-10: F32, F33)

ICD-10International Classification of Diseases, 10th revisionpoppopulation

### Outcomes

Health outcomes were coded as binary (yes/no) variables for the follow-up period of 2001–2018 unless otherwise stated. We examined eight principal outcomes under five headings: (1) all causes: death, hospitalisation; (2) CVD: death, hospitalisation, prescription; (3) cancer: death, hospitalisation; (4) mental health: prescription medication for hypnotics and anxiolytics and for antidepressants (follow-up 2009–2018); and (5) traffic casualty: traffic casualty hospitalisation. Table 2 provides further details of the diagnostic codes (International Classification of Diseases, 9th and 10th revisions (ICD-9 and ICD-10)) and prescription ((British National Formulary (BNF)) codes used to define each outcome variable. Most of the outcomes (hospitalisations and deaths for all causes, cancer, CVD) were selected based on previous studies^7^; prescriptions data enabled examination of an additional CVD outcome and a mental health outcome (anxiety and depression); and the risk of being a traffic casualty is relevant to active commuting.^22^

**Table 2:** Outcome variables and definitions

Outcomes	ICD codes	Exposure period
All causes		
Mortality from all causes	Any diagnosis	2001–2018
Any hospital admission	Any diagnosis	2001–2018
Cardiovascular disease (CVD)		
CVD mortality	ICD-10 codes: I00-I99 (primary diagnosis)	2001–2018
CVD hospital admission	ICD-10 codes: I00-I99 (primary diagnosis)	2001–2018
CVD prescription	BNF codes: 2.2, 2.4, 2.5, 2.6, 2.8.1, 2.8.2, 2.9, 2.12	2009–2018
Cancer		
Cancer mortality	C00-C97 (primary diagnosis)	2001–2018
Cancer hospital admission	C00-C97 (primary diagnosis)	2001–2018
Mental health		
Mental health prescription (for anxiety and depression)	BNF codes: 4.1 (hypnotics and anxiolytics) and 4.3 (antidepressant drugs)	2009–2018
Traffic casualties		
Hospital admission due to injury in a transport incident	ICD-10 codes V01-V99 (any position)	2001–2018

BNFBritish National FormularyICD-10International Classification of Diseases, 10th revision

### Statistical analysis

Descriptive statistics were used to compare the proportion of participants in each covariate category by each mode of travel (see table 1). Cox proportional hazard models were used to estimate the association between mode of travel and each health outcome occurring between 2001 and 2018 unless otherwise stated. The reference category for all analyses was non-active commuters (those commuting by public transport or motor vehicle). Models were adjusted for the covariates listed above and, additionally, with and without car ownership, a potential confounder. The results were similar and the final model results presented are those for the models excluding adjustment for car ownership. The assumption of proportional hazards was assessed based on tests of Schoenfield residuals. If variables failed to meet this assumption, they were stratified within the model. The R version 3.6.3 (R Core Team, 2020)^23^ and the ‘survival package’^24^ were used for all analysis.

## Results

### Cohort description

We followed 82 297 participants from the 2001 Scottish Longitudinal Study until 2018. Over the follow-up period: a total of 4276 participants died (5.2% of the cohort) and, of these, almost half died of cancer (2023, 2.5%); 52 804 participants (64.2%) had a hospital admission and, of these, 9663 (11.7%) had a hospital admission for CVD, 5939 (7.2%) were hospitalised for cancer, and 2668 (3.2%) were hospitalised after a traffic collision; 31 666 participants (38.5%) received a CVD related prescription in the period 2009–2018, and 33 771 participants (41%) had a prescription for poor mental health over the same period.

Table 1 shows descriptive statistics for the covariates by commuting mode. Compared with non-active commuters, pedestrian commuters were more likely to be female, younger, shift workers, commute shorter distances, and live in a city. They were less likely to have dependent children and generally had a lower socioeconomic position (for example, in terms of educational qualifications, occupation, home ownership, and likelihood of living in overcrowded households). Compared with non-active commuters, cyclist commuters were more likely to be male, younger, shift workers and live in a city, and were less likely to be homeowners or carers.

### Model results

**Figure 1:**
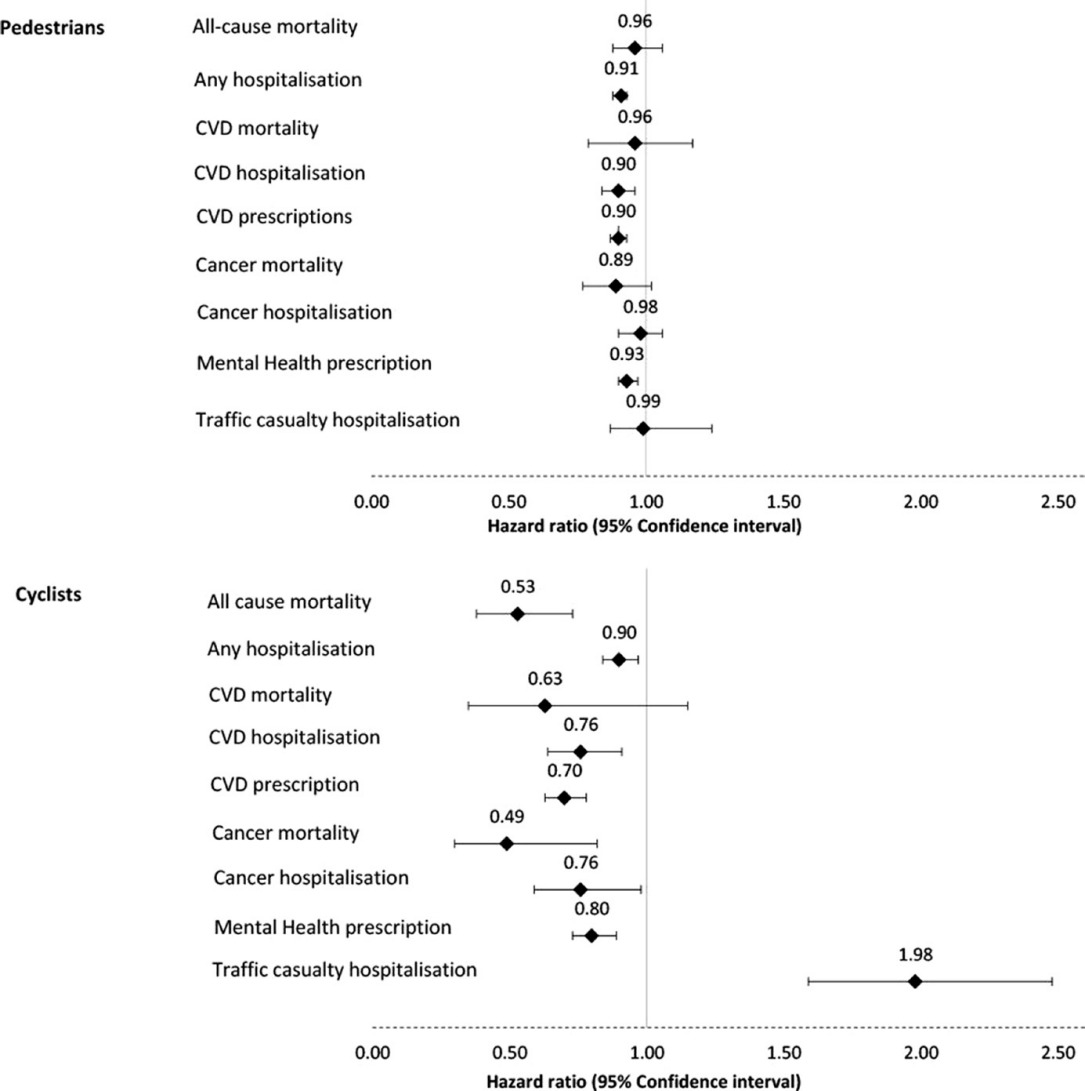
Health outcomes by mode of travel to work or study in Scotland. Hazard ratios and 95% confidence intervals, active modes compared to non-active travel. CVD, cardiovascular disease.

The main results are detailed in table 3 and illustrated in figure 1. Cyclist commuters, compared with non-active commuters, were associated with a lower risk of: all-cause mortality (HR 0.53, 95% CI 0.38 to 0.73), any hospitalisation (HR 0.90, 95% CI 0.84 to 0.97), CVD hospitalisation (HR 0.76, 95% CI 0.64 to 0.91), receiving a CVD related prescription (HR 0.70, 95% CI 0.63 to 0.78), cancer mortality (HR 0.49, 95% CI 0.30 to 0.82) and cancer hospitalisation (HR 0.76, 95% CI 0.59 to 0.98), and having a prescription for a mental health condition (HR 0.80, 95% CI 0.73 to 0.89). There was no clear evidence of an association between cyclist commuters and CVD mortality (HR 0.63, 95% CI 0.35 to 1.15). Cyclist commuters, compared with non-active commuters, were associated with an increased risk of hospitalisation after a traffic collision (HR 1.98, 95% CI 1.59 to 2.48), although this was a relatively rare event (83 hospitalisations over 18 years).

**Table 3:** Risk of various adverse health outcomes for active (cyclist, pedestrian) commuters versus non-active commuters, 2001–2018

	Outcome	Category	n (%)	HR (95% CI)
All causes	All-cause mortality^a^	Non-active	3620 (5.2)	1.00
		Active: cyclist	38 (2.8)	0.53 (0.38 to 0.73)
		Active: pedestrian	618 (5.3)	0.96 (0.88 to 1.06)
	Any hospitalisation^b^	Non-active	45 026 (64.9)	1.00
		Active: cyclist	770 (56.5)	0.90 (0.84 to 0.97)
		Active: pedestrian	7708 (60.6)	0.91 (0.88 to 0.93)
CVD	CVD mortality^c^	Non-active	824 (1.2)	1.00
		Active: cyclist	11 (0.8)	0.63 (0.35 to 1.15)
		Active: pedestrian	138 (1.2)	0.96 (0.79 to 1.17)
	CVD hospitalisation^d^	Non-active	8343 (12.0)	1.00
		Active: cyclist	125 (9.2)	0.76 (0.64 to 0.91)
		Active: pedestrian	1195 (10.3)	0.90 (0.84 to 0.96)
	CVD prescription*^e^	Non-active	27 185 (39.2)	1.00
		Active: cyclist	363 (26.6)	0.70 (0.63 to 0.78)
		Active: pedestrian	4118 (35.6)	0.90 (0.87 to 0.93)
Cancer	Cancer mortality^f^	Non-active	1734 (2.5)	1.00
		Active: cyclist	15 (1.1)	0.49 (0.30 to 0.82)
		Active: pedestrian	274 (2.4)	0.89 (0.77 to 1.02)
	Cancer hospitalisation^g^	Non-active	5081 (7.3)	1.00
		Active: cyclist	63 (4.6)	0.76 (0.59 to 0.98)
		Active: pedestrian	795 (6.9)	0.98 (0.90 to 1.06)
Mental health	Mental health prescription*^h^	Non-active	28 599 (41.2)	1.00
		Active: cyclist	414 (30.0)	0.80 (0.73 to 0.89)
		Active: pedestrian	4758 (41.2)	0.93 (0.90 to 0.97)
Traffic casualties	Traffic casualty hospitalisation^i^	Non-active	2225 (3.2)	1.00
		Active: cyclist	83 (6.1)	1.98 (1.59 to 2.48)
		Active: pedestrian	360 (3.1)	0.99 (0.87 to 1.12)

Variables not satisfying the proportional hazards assumption were stratified and are; agea, b, d, e, f, g, h, i, dependent childrenb, d, e, h, i, distance to workb, g, highest qualificatione, h, household crowdingd, housing tenurea, b, d, h, National Statistics-Socioeconomic Classificationb, c, h, i, pre-existing health conditiona, b, d, e, f, g, sex b, c, d, e, g, h, i, shift workera, h, and urban-rural residence.b, e Based on Cox’s proportional hazards multivariable regression models.

*The period of follow-up for the prescription outcomes was 2009–2018.

CVDcardiovascular disease

Pedestrian commuters, compared with non-active commuters, were associated with a lower risk of any hospitalisation (HR 0.91, 95% CI 0.88 to 0.93), CVD hospitalisation (HR 0.90, 95% CI 0.84 to 0.96), receiving a CVD related prescription (HR 0.90, 95% CI 0.87 to 0.93), and having a prescription for a mental health condition (HR 0.93, 95% CI 0.90 to 0.97). There was no clear evidence of an association between pedestrian commuters and all-cause mortality (HR 0.96, 95% CI 0.88 to 1.06), CVD mortality (HR 0.96, 95% CI 0.79 to 1.17), cancer mortality (HR 0.89, 95% CI 0.77 to 1.02) and cancer hospitalisation (HR 0.98, 95% CI 0.90 to 1.06), or hospitalisation after a traffic collision (HR 0.99, 95% CI 0.87 to 1.12).

## Discussion

### Overall findings

This is the first study to provide direct evidence of the association between active and non-active commuting and health outcomes over a long period for Scotland. The study identified clear and consistently lower risks of adverse health outcomes among active commuters, especially cyclists. Compared with non-active commuters, cyclists had 47% lower risk of death from any cause, 10% lower risk of any hospitalisation, 24% lower risk of CVD hospitalisation and 30% lower risk of receiving a CVD related prescription, 24% lower risk of cancer hospitalisation and 51% lower risk of cancer death, and a 20% lower risk of receiving a mental health related prescription. Pedestrian commuters, compared with non-active commuters, had a 9% lower risk of any hospitalisation, 10% lower risk of CVD hospitalisation or of receiving a CVD related prescription, and 7% lower risk of receiving a mental health related prescription. However, cycle commuters were twice as likely as non-active commuters to be hospitalised due to a traffic collision.

### Comparison to previous research

Previous research has associated commuting by bicycle with a lower risk of CVD, cancer, and all-cause mortality, and walking commuting with a lower risk of CVD, compared with non-active commuting (car or public transport)^7^, findings which are similar to our study’s results. A UK Census-based longitudinal study with participant follow-up over 25 years showed similar but lower associated reductions in all-cause, CVD and cancer mortality and in cancer incidence for cyclist commuters compared with commuters using a private motorised vehicle. Additionally, pedestrian commuting was associated with 7% lower risk of cancer incidence.^25^ There were differences between our study and this previous investigation. In the latter, three waves of English and Welsh census data were used; active commuters were compared with commuters using private motorised vehicles, whereas our reference group was all non-active commuters (ie, car and public transport); there was a longer maximum follow-up period than in our study; and the study controlled for slightly different covariates. These differences may have contributed to the lower benefits associated with active commuting.

Similar to our study, commuting by bicycle has previously been associated with a higher risk of hospital admission after a transport related incident in comparison to non-active commuting modes,^26^ and consistent with our findings, this study also showed that commuters who cycled to work had a lower risk of CVD, cancer, and death compared with non-active commuters. Other studies have confirmed that the health benefits of cycling are much greater than the risk of injuries.^27^

In previous research, a positive association has been shown between active travel and good mental health,^28^ and more specifically between active commuting and psychological well-being.^8^ Another study showed that cycling commuters reported lower sickness absence and better mental health.^29^ In contrast to these studies in which mental health was self-assessed, the mental health outcome used in our study is based on whether a participant has been prescribed a medication to treat anxiety and depression. The mental health benefits of both walking and cycling to work demonstrated in our study are notable, particularly given the high proportion of cohort participants (41%) who were prescribed medication for poor mental health.

The significant contribution that active commuting makes to total physical activity may explain the positive associations active travel has had with health outcomes. One study found a 44% increase in physical activity levels in individuals who walked to work compared with those who travelled by car.^30^ Another Scottish study estimated that 46.5% of all active commuters in 2001 met a daily target of 30 min of moderate intensity activity from their commute alone.^19^ Our study did not account for physical activity unrelated to active travel and this may partially explain the larger reduction in all-cause mortality risk associated with cyclist commuting compared with other studies.^3 7^

Active commuting has clear health benefits and can be an effective way to accommodate physical activity into everyday working life.^4^ However, trends toward fewer commuting journeys,^31^ greater home working,^32^ and growing support for more flexible working practice—‘hybrid working’—have been accelerated by the COVID-19 pandemic^33 34^ and could reduce the opportunity for active travel to work. Nevertheless, active travel is a safe and healthy activity that was supported during the pandemic,^35^ leading to calls that it should be promoted by more investment in the post-COVID recovery period.^36 37^

Many governments and cities are now focused on making a modal shift from car use towards more sustainable modes of transportation, such as walking and cycling, to cut carbon and pollutant emissions and to improve liveability.^38^ In Scotland there are similar commitments,^16 18^ and clear evidence of the health benefits of active travel provides an additional reason to support sustained active travel investment.

### Strengths and weaknesses

The main exposure variable is limited as it is recorded only at one point in time, 2001, and respondents may have subsequently changed their method of commuting or stopped commuting. The Census does not capture multi-modal trips and so there may be overlap between active and non-active commuters which could underestimate the association between active travel and health. Additionally, we could not model a dose-response relationship with the available exposure data, although we have controlled for commuting distance. Previous research has demonstrated that more intense forms of active travel have stronger associations with physical health, such as cycling or walking more than 6 miles (10 km) per week.^7^ The removal of records with missing covariate data may have introduced unknown bias, although the distribution of missingness was similar across modes of travel.

Due to the limitations of the SLS, we were unable to adjust for some potential confounders, such as income or body mass index at baseline, we were not able to account for time-varying confounding and excluded individuals with missing covariate data. Additionally, active commuting may be associated with other forms of physical activity that contribute to total physical activity levels.^39^ However, we were unable to adjust for other forms of physical activity, another limitation of the dataset used. This may have led to an overestimation of the effect estimates, particularly for cyclist commuters who have been shown to have higher levels of overall physical activity than other commuters.^7^ Nonetheless, previous research has demonstrated that beneficial associations between active travel and health remained after adjustment for other physical activity.^3^

Prescription-based outcomes could only be followed up from 2009 onwards as earlier years were not available. Our study has shown a positive association between active commuting and one measure of mental ill-health, but data were unavailable to measure any potential association with mental well-being.

We do not have information on the severity of traffic casualties, although an injury requiring hospital admission is likely to be serious. Minor injuries, not requiring hospital treatment, will have been missed and under-reporting is high for cyclists compared with other modes.^40^ So the risk of injury after a traffic collision is likely to be an underestimate.

The strengths of this study lie in the use of the SLS. Compared with another UK study based on UK Biobank data,^7^ our study had a longer follow-up period (18 years compared with a median of 5 years) and a wider age range of participants (aged 16–74 compared with aged 40–49). The participants in our study are from a Census sample, representative of the Scottish general population, which is not subject to healthy respondent bias that is inherent in surveys.^41^

We designed our study to compare cyclist commuter outcomes and pedestrian commuter outcomes with the non-active commuter group, separately. We did not aim to compare directly cyclist commuter outcomes to pedestrian commuter outcomes, as doing so would have excluded non-active commuters from the sample (reducing its size and representativeness), introduced issues with the interpretation and may have introduced collider bias.^42^

The prospective study design and adjustment for pre-existing health conditions allowed us to address reverse causality which was highlighted as a limitation in previous research,^28^ although residual confounding from undiagnosed conditions presenting early in the follow-up period cannot be ruled out. The use of a large sample of census data linked to national health records, which have quality assured coding, has reduced the risk of attrition bias and improved the reliability of the outcome measures. We measured a range of health outcomes, including mental health via innovative use of prescription data, thereby providing a broad assessment of the positive impacts of active commuting. The study provides important policy-relevant evidence for Scotland, the UK and internationally.

### Policy context

Health, environmental and planning policies in Scotland have become progressively aligned in support of active travel.^16^ Funding for active travel has increased substantially in recent years and is set to rise further to £320 million (€375 million, US$400 million)) per annum, representing £58 (€68, US$73) per head of population, in 2024/25.^18^ Yet the potential health benefits that accompany active travel are often assumed or implied, but without specific evidence. These findings provide direct evidence of the health benefits of active commuting in a Scottish context, and add to previous modelling which suggested substantial health and economic benefits accrued from active commuting at a population level.^19^ Given the substantial planned investment in active travel in Scotland, our finding that cyclist commuters have twice the risk of being a road traffic casualty compared with non-active commuters reinforces the need for safer cycling infrastructure.

## Conclusion

This study strengthens the evidence that active commuting has population-level health benefits and can contribute to reduced morbidity and mortality. That cyclist and pedestrian commuting is associated with lower risks of being prescribed medication for poor mental health is an important finding. These findings provide direct evidence of the health benefits of active commuting in a Scottish context, supporting current policy. This study has wider global relevance to efforts to reduce carbon emissions and to shift to more active and sustainable travel modes.

## Data Availability

All data relevant to the study are included in the article.
